# Deficiency of DICER reduces the invasion ability of trophoblasts and impairs the pro‐angiogenic effect of trophoblast‐derived microvesicles

**DOI:** 10.1111/jcmm.14917

**Published:** 2020-03-21

**Authors:** Li Tang, Ming Yang, Lang Qin, Xiaoliang Li, Guolin He, Xinghui Liu, WenMing Xu

**Affiliations:** ^1^ Department of Obstetrics/Gynecology West China Second University Hospital Sichuan University Chengdu China; ^2^ The Joint Laboratory for Reproductive Medicine of Sichuan University‐The Chinese University of Hong Kong West China Second University Hospital Sichuan University Chengdu China; ^3^ The Key Laboratory of Birth Defects and Related Diseases of Women and Children Ministry of Education West China Second University Hospital Sichuan University Chengdu China; ^4^ Reproductive Endocrinology and Regulation Laboratory West China Second University Hospital Sichuan University Chengdu Sichuan China

**Keywords:** angiogenesis, COL1A2, DICER, invasion, microvesicle, miR‐16‐2, spiral artery remodelling, trophoblast

## Abstract

DICER is a key rate‐limiting enzyme in the canonical miRNAs biogenesis pathway, and DICER and DICER‐dependent miRNAs have been proved to play essential roles in many physiological and pathological processes. However, whether DICER is involved in placentation has not been studied. Successful spiral artery remodelling is one of the key milestones during placentation, which depends mostly on the invasion of trophoblasts and the crosstalk between trophoblasts and endothelial cells. In the present study, we show that DICER knockdown impairs the invasion ability of both primary extravillous trophoblasts (EVT) and HTR8/SVneo (HTR8) cell lines. The decreased invasion of HTR8 cells upon DICER knockdown (sh‐Dicer) was partly due to the up‐regulation of miR‐16‐2‐3p, which led to a reduced expression level of the collagen type 1 alpha 2 chain (COL1A2) protein. Moreover, microvesicles (MVs) can be secreted by HTR8 cells and promote the tube formation ability of human umbilical cord vein endothelial cells (HUVECs). However, conditioned medium and MVs derived from sh‐Dicer HTR8 cells have an anti‐angiogenic effect, due to reduced angiogenic factors and increased anti‐angiogenic miRNAs (including let‐7d, miR‐1‐6‐2 and miR‐15b), respectively. In addition, reduced protein expression of DICER is found in PE placenta by immunoblotting and immunohistochemistry. In summary, our study uncovered a novel DICER‐miR‐16‐2‐COL1A2 mediated pathway involved in the invasion ability of EVT, and DICER‐containing MVs mediate the pro‐angiogenic effect of trophoblast‐derived conditioned medium on angiogenesis, implying the involvement of DICER in the pathogenesis of PE.

## 1 INTRODUCTION

Remodelling of uterine spiral artery (SA), an end artery of the uteroplacental circulation, is the process of disruption of normal structure of SA by invading trophoblasts, to supply enough maternal blood to the placenta during the first trimester of pregnancy. Failure of spiral artery remodelling often results in reduced placental perfusion and pregnancy complications, like preeclampsia (PE).^1^, ^2^ Successful SA remodelling mostly depends on the invasion of extravillous trophoblasts (EVT) and the interaction between EVT and endothelial cells of the vessel.^3^ Trophoblasts are specialized cells in the placenta, and there are three major sub‐populations of trophoblasts during placentation: cytotrophoblasts (CTB), EVT and syncytiotrophoblasts (STB). Impaired invasion of EVT is believed to be involved in the pathogenesis of PE. Therefore, several studies have been conducted to investigate how stresses like hypoxia, inflammation and specific molecules influence the migration and invasion of EVT.^4^, ^5^

Among the latter, microRNAs (miRNAs) emerged as key players. miRNAs are single‐stranded oligonucleotide guide RNA sequences with 22‐25 bases which, by repressing target gene expression at the post‐transcriptional level, regulate various physiological and pathological process, including the behaviour of trophoblasts.^6^ DICER is a key enzyme in miRNA biogenesis, being responsible for the processing of pre‐miRNAs into mature miRNAs.^7^ Although the effects of individual miRNAs and their corresponding target genes on the invasion of EVT have been extensively studied, the role of DICER in placentation, and more specifically in trophoblast function, is still obscure.

Extracellular vesicles (EVs) are important mediators of intercellular communication and were recently shown to play key roles in placentation. EVs are membrane vesicles that are secreted from various cell types into the extracellular space, carrying biomolecules of donor cells into recipient cells, like proteins, RNAs and lipids. According to the diameter, EVs are subdivided into exosomes, microvesicles and apoptotic bodies.^8^, ^9^ Abundant evidence has proved that trophoblast‐derived EVs have several roles during pregnancy, which include immune modification and establishment of foetal‐maternal circulation.^10^, ^11^, ^12^ So far, the interaction between EVT and the endothelial cells in the spiral artery has been seldomly studied, unlike the invasion ability of EVT, despite being a critical factor during spiral artery remodelling.

In this study, we investigated the role of DICER in EVT function in the process of spiral artery remodelling and whether DICER is involved in the pathogenesis of PE. Results show that DICER knockdown inhibited the invasion ability of HTR8 cells through up‐regulation of miR‐16‐2 expression, which targets collagen type 1 alpha 2 chain (COL1A2). In addition, DICER is shown to be critical for mediating the pro‐angiogenetic effect of trophoblast‐derived microvesicles (MVs) on endothelial cells by affecting the angiogenic balance of MVs. Furthermore, the expression of DICER is decreased in PE placenta, implying that DICER plays important roles in placentation and that aberrant expression of DICER may be involved in the pathogenesis of PE.

## 2 MATERIALS AND METHODS

### 2.1 Patients and tissue collection

Clinical samples were collected from women who visited the Department of Obstetrics and Gynecology, West China Second University Hospital of Sichuan University in Chengdu, China. All study participants provided written informed consent. The study was approved by the Ethical Review Board of West China Second University Hospital, Sichuan University. In this study, placenta tissues were collected from 10 normotensive pregnancies, 6 PE cases and 6 severe PE cases. PE was diagnosed in the cases where the blood pressure was ≥140/90 mm Hg after 20 weeks of pregnancy, and the urinary protein excretion was of 300 mg or more in 24 hours. sPE was diagnosed in the cases where the blood pressure was ≥160/110 mm Hg and the urinary protein excretion was of 3 g or more in 24 hours according to previously published reports.^13^, ^14^ All types of pregnancy related diseases were excluded from the control group. The patient characteristics are summarized in Table S1. Placenta tissues were cut after delivery and were stored at −80°C or fixed in 4% paraformaldehyde (PFA) for further analysis. For isolating human umbilical cord vein endothelial cells (HUVECs), a segment of the umbilical cord was cut at delivery of a normal pregnancy. For isolating human first‐trimester trophoblast cells, chorionic villi were obtained from healthy women undergoing suction termination of pregnancy (8‐12 weeks) for non‐medical reasons.

### 2.2 Isolation of human first‐trimester trophoblast cells

The trophoblast cells were isolated from human first‐trimester villi as described previously with some modifications.^15^ Briefly, villous tissue was cut into small pieces and digested with 0.125% trypsin and 0.04% DNAse I (Sigma). The cells were pelleted after filtration by a cell filter. Then, cells were washed and resuspended in 1× HBSS, and fractionated on Percoll‐HBSS density medium by centrifugation. Cells that sedimented at densities between 30% and 50% were collected. The isolated trophoblasts were cultured in fibronectin‐coated plates with Ham's F‐12K medium supplemented with 10% FBS and 2 mmol/L glutamine (GlutaMAX™ Supplement, Thermo) and incubated in 5% CO_2_ at 37°C. Anti‐CK7 (381105, Zen‐Bio) and anti‐HLA‐G (ab7758, Abcam) antibodies were employed to identify the purity of EVT.

### 2.3 Plasmid construct

Addgene plasmids were used for lentivirus packaging, including pSicoR human Dicer2 (14764), scramble shRNAs (1864‐LV), psPAX2 (12260) and pMD2.G (12259). The COL1A2 overexpression plasmid was purchased (sc126717, OriGene). The 3′‐UTR region of COL1A2 was amplified by PCR. The PCR product was cloned downstream of the dual luciferase reporter psiCHECK‐2 vector (Promega) by Trelief™ SoSoo Cloning Kit (TsingKe Biotech) to form a wild‐type plasmid. The mutant vector was generated by replacing the ATATT bases of seed region into the ACACC bases. Primers for PCR are listed in Table S2.

### 2.4 Cell lines and transfection

The human HTR8/SVneo and HEK 293T cell lines were cultured in Dulbecco's modified Eagle medium (DMEM) supplemented with 10% FBS at 37°C and 5% CO_2_. Lipofectamine 3000 (Invitrogen) was used for all transfections according to the manufacturer's instruction. si‐Dicer RNA (CATTGATCCTGTCATGGAT), si‐Col1a2 RNA (GTGGATACGCGGACTTTGT), miRNA mimics and miRNA inhibitors were purchased from RiboBio.

### 2.5 Conditioned medium preparation and isolation of EVs and MVs

Conditioned medium (CM) was collected from nearly confluent, untreated HTR8 cells, sh‐scr or sh‐Dicer HTR8 cells, 24 hours after culturing in serum‐free DMEM. Collected CM was then centrifuged at 1500 *rcf* for 10 minutes at 4°C, to remove cell debris. EVs were pelleted from CM at 100 000 *rcf* for 2 hours at 4°C, and microvesicles (MVs) were pelleted from CM at 15 000 *rcf* for 1 hours at 4°C, as previously described.^16^ Pelleted EVs and MVs were resuspended in PBS and stored at −20°C, or lysed in protein extraction lysis buffer or TRIzol reagent.

### 2.6 Nanoparticle tracking analysis (NTAs) for exosomes—NTA

Nanoparticle tracking analysis (NTA) was conducted using the Malvern Zetasizer Nano ZS90. EVs were collected from CM and then analysed by NTA version 2.1, and all analysis settings were kept constant within each experiment. The capture and analysis settings were determined manually according to the manufacturer's instructions.

### 2.7 Flow cytometry analysis

The concentration of purified MVs diluted in PBS was analysed by flow cytometry using a GUAVA EASYCYTE HT FLOW CYTOMETER (Millipore). A gate was established to include the centralized events. The concentration of MVs was analysed by GuavaSoft Software in a medium flow option.

### 2.8 Electroporation

DICER antibody was loaded onto MVs as previously described.^17^ Briefly, purified MVs were resuspended in electroporation buffer with a protein concentration equal to 0.3 µg/µl. We added 10 µg of DICER antibody (ab14601, Abcam) or 10 µg of rabbit IgG (ab172730, Abcam) into 500 µl of diluted MVs, and the mixture was loaded into electroporation cuvettes, with a gap width of 0.4 cm (Bio‐Rad). The electroporation was performed by the Gene Pulser Xcell™ Electroporator (Bio‐Rad) using the square wave protocol. Then, electroporated MVs were washed in PBS with 1% BSA. Pelleted MVs were resuspended in PBS again, and the same amount of MVs was assayed with the BCA kit before treating HUVECs.

### 2.9 Tube formation assay

Matrigel (Corning) was used to assess the formation of capillary‐like structures as previously described.^18^ HUVECs were collected from umbilical cords by collagenase perfusion. For observing the effect of CM or MVs on angiogenesis, HUVECs were resuspended in CM supplemented with 2% FBS or DMEM containing a certain amount of MVs supplemented with 2% FBS and then plated on top of Matrigel. Tube formation was visualized under a bright‐field microscope 8 hours after implantation. For better visualization, HUVECs in Figure 5G and H were stained with crystal violet before taking pictures. The total tube length of 3 random microscopic fields was quantified by the NIS‐Elements D 3.1 software (https://nis-elements-d.software.informer.com/3.1).

### 2.10 Luciferase assay

Firefly luminescence and Renilla luminescence of wild‐type and mutant psiCHECK2‐COL1A2 vector in HTR8/Svneo cells were assessed by Dual‐Glo^®^ Luciferase Assay System (Promega) 24 hours after transfection of miRNA mimic or inhibitor, according to the manufacturer's instructions.

### 2.11 Matrigel invasion assay

The invasion of EVT across Matrigel was evaluated in transwell chambers (Corning). For transfected cells, invasion assay was performed 24 hours after transfection. Briefly, the transwell inserts were pre‐coated with Matrigel and were placed in a cell incubator for coagulation. Trophoblast cells suspended in serum‐free medium were plated in the upper chamber. The lower chamber was added with DMEM with 30% FBS. The cells were then incubated at 37.0°C for 48 hours. The cells on the lower side of the filter were then stained with 0.5% crystal violet after the non‐invading cells were removed from the upper surface. Transwell fields were photographed using a light microscope (Olympus). ImageJ software (http://rsb.info.nih.gov/nih-image/) was used to estimate the cell density.

### 2.12 miRNA sequencing

Total RNA was extracted using mirVana miRNA Isolation Kit (Ambion) according to the manufacturer's protocol. We used 1 µg total RNA per sample for the small RNA library construction using TruSeq Small RNA Sample Prep Kits (Cat. No. RS‐200‐0012, Illumina) following the manufacturer's recommendations. The RNA library quality was assessed on the Agilent Bioanalyzer 2100 system using DNA High Sensitivity Chips. The libraries were finally sequenced using the Illumina HiSeq X Ten platform. Small RNA sequencing and analysis were conducted by OE Biotech Co., Ltd..

### 2.13 Isolation of RNA and reverse‐transcription real‐time PCR

Total RNA was isolated using TRIzol (Invitrogen), and cDNA was synthesized by a miRNA‐specific reverse‐transcription primer for miRNA (RiboBio) or random hexamers for mRNA by RT Easy™ I Kit (Foregene). The cDNA of miRNA was amplified with a specific forward primer and a universal reverse primer (RiboBio). Quantitative real‐time PCR was performed using an Applied Biosystems 7500 Sequence Detection System with SYBR green chemistry. Primers for qPCR are listed in Table S2.

### 2.14 Immunofluorescence staining

EVT were plated on coverslips and fixed with 4% PFA, then permeabilized with 0.3% Triton X‐100, and finally blocked with 5% BSA. Then, the cells were incubated with primary antibodies against CK7 (ET1609‐62; HuaBio; 1:300) and HLG‐A (ab7758; Abcam; 1:200) at 4°C overnight. After incubation with DyLight 488‐ or DyLight 594‐labelled secondary antibodies (1:1000, Thermo Fisher), the nuclei were counterstained with DAPI (Sigma). Images were acquired using a laser scanning confocal microscope (Olympus).

### 2.15 Immunohistochemistry (IHC) assays

Fixed placental tissue was dehydrated, embedded in paraffin and sectioned to 5‐µm sections. Tissue sections were deparaffinized, rehydrated and retrieved by boiling in 0.01 mol/L sodium citrate. The following procedures were performed by SPlink Detection Kit and DAB Kit (ZSGB‐Bio) according to the manufacturer's instruction. Briefly, the tissue sections were treated with 3% H_2_O_2_ and goat serum in sequence. Then, the tissue sections were incubated with a primary antibody against DICER (506021; Zen‐Bio; 1:50) or rabbit IgG (ab172730, Abcam; 1:50) at 4°C overnight, followed by incubation with a secondary antibody. The immunocomplexes were visualized with DAB solution, and the nuclei were stained with haematoxylin. The slides were dehydrated with ethanol, sealed with coverslips and imaged under a light microscope (Olympus).

### 2.16 Western blot analysis

Total protein from the cells or tissues was extracted using radioimmunoprecipitation assay (RIPA) buffer with a protease inhibitor cocktail (Roche). Lysates were denatured prior to eletrophoresis and then transferred to PVDF membranes (Millipore). The immunoreactive signals were visualized using enhanced chemiluminescence reagents (ECL, Pierce). The following antibodies and dilutions were used: anti‐DICER (506021, Zen‐Bio; 1:500), anti‐COL1A2 (505786, Zen‐Bio; 1:1000), anti‐MMP9 (ET1704‐69, HuaBio; 1:1000), anti‐MMP2 (220780, Zen‐Bio; 1:1000) and anti‐VEGFA (ER30607, HuaBio; 1:500).

### 2.17 Enzyme‐linked immunosorbent assay (ELISA)

Human VEGF ELISA Kit (EHC108.96, NeoBioscience) was used to determine VEGFA levels in CM according to the manufacturer's instructions. Concentrations were calculated by a standard curve, with *R* ^2^ above .99.

### 2.18 Human angiogenesis antibody array

The levels of 55 angiogenesis‐associated factors in CM were detected by Human Angiogenesis Antibody Array Kit (ARY007, R&D) according to the manufacturer's instruction. In brief, 200 µl samples were mixed with the reconstituted Detection Antibody Cocktail. After incubation, the membranes were treated with 1.5 mL of the sample/antibody mixture overnight at 4°C on a rocking platform shaker followed by three washes. Then, the membrane was incubated with streptavidin‐horseradish peroxidase solution followed by three washes. Finally, the signals were generated by exposure to an ECL chemiluminescent substrate supplied in the kit.

### 2.19 Statistics

All data were analysed using Prism 6 (GraphPad), and the results are presented as mean ± SEM (standard error of mean), from at least 3 independent experiments. Unpaired two‐tailed Student's *t* test was used to assess 2 independent groups. One‐way ANOVA was used to test multigroup comparisons with post hoc Dunnett's multiple comparison test. In figures, ns stands for not significant and asterisks indicate the *P*‐value as follows: *P < .05, **P < .01 and ***P < .001.

Data availability: Data on miRNA sequencing analysis described here are available on ‘PRJNA534361’. All relevant data that support the findings of this study are available from the corresponding author upon reasonable request.

## 3 RESULTS

### 3.1 DICER knockdown impairs the invasion ability of primary EVTand HTR8/SVneo cells

**Figure 1:**
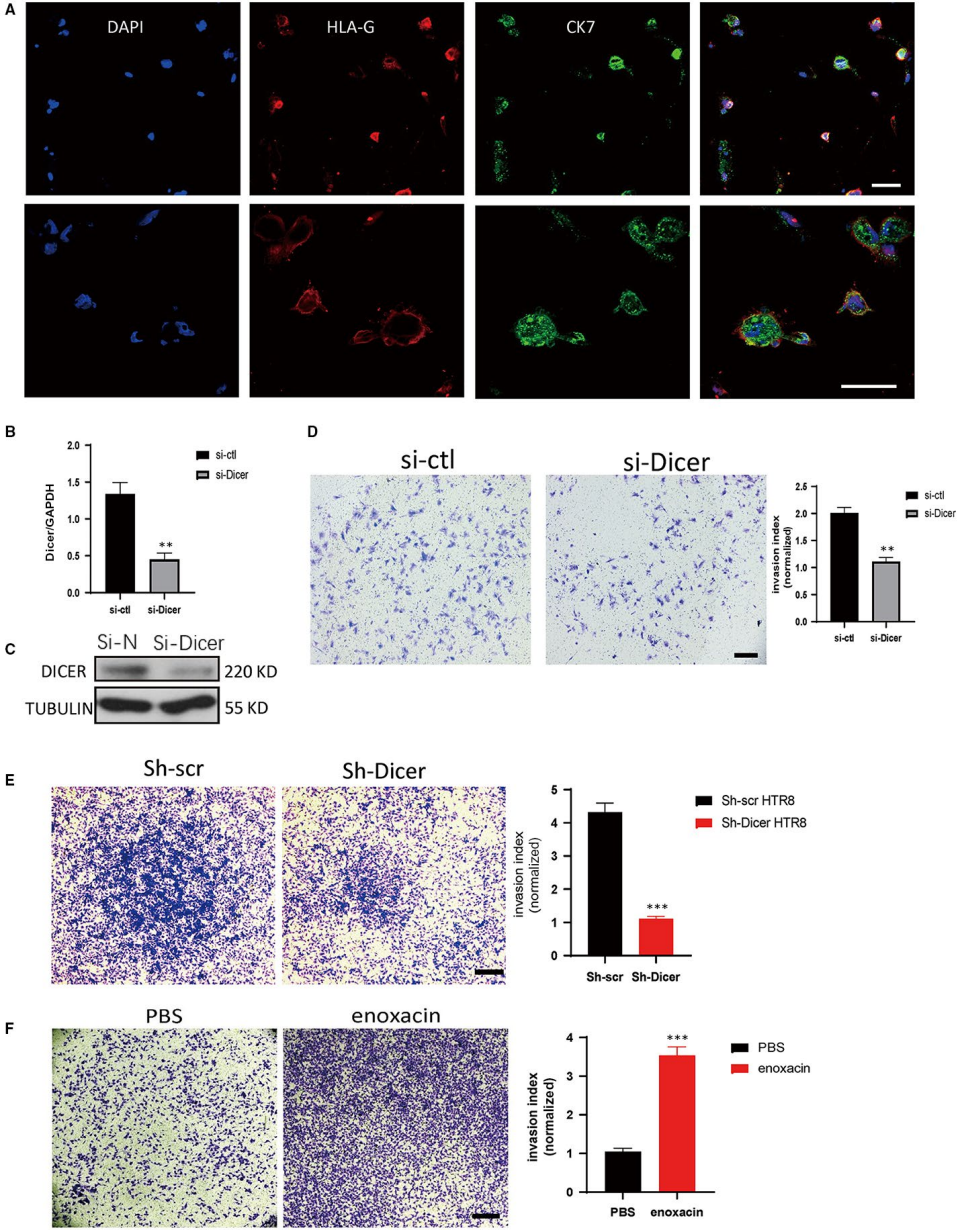
DICER knockdown impairs the invasion ability of both primary EVT and HTR8 cell line. A, Double staining of CK7 and HLA‐G by immunofluorescence to identify isolated human primary first‐trimester trophoblast (upper, 20×; lower, 40×). Scale bar = 40 µm. B, DICER mRNA levels in primary EVT relative to GAPDH determined by qPCR following transfection of control siRNA (si‐ctl) or DICER siRNA (si‐Dicer). The mean ± SEM is shown. **P < .01 (n = 3). C, Representative immunoblot of DICER in primary EVT following transfection of si‐ctl or si‐Dicer RNA. D, Invasion assay of HTR8 cells transected with si‐ctl or si‐Dicer RNA (top). Scale bar = 100 µm. Quantification of cell number at the outside membrane of transwell insert (bottom). The mean ± SEM is shown. **P < .01 (n = 3). E, Invasion assay of HTR8 cells transected with sh‐scr or sh‐Dicer LVs (top). Scale bar = 200 µm. Quantification of cell number at the outside membrane of transwell insert (bottom). The mean ± SEM is shown. ***P < .001 (n = 3). F, Invasion assay of HTR8 cells with the treatment of 10 mmol/L enoxacin. Scale bar = 200 µm. Quantification of cell number at the outside membrane of transwell insert (bottom). The mean ± SEM is shown. ***P < .001 (n = 3)

To identify the specific roles of DICER in placentation, here we focus on how DICER affects the invasion ability of trophoblasts. Human primary first‐trimester trophoblasts were isolated and identified by two EVT‐specific markers, CK7 and HLA‐G (Figure 1A).^19^, ^20^ The invasion ability of EVT was significantly decreased after DICER knockdown (Figure 1B‐D). Since primary trophoblasts rapidly cease proliferation in vitro, which is adverse to study the downstream signalling changes following gene manipulation, the HTR8/SVneo (HTR8) cell line is used to study the mechanism of how DICER affects the invasion of trophoblasts.^21^ Consistently, the invasion ability of DICER knockdown HTR8 cells mediated by a lentiviral shRNA (sh‐Dicer HTR8) was remarkably decreased, compared to that of sh‐scr HTR8 (Figure 1E; Figure S1A‐B). DICER knockdown plasmid was from and well applied in Jacks Lab.^22^ The small‐molecule enoxacin promotes the up‐regulation of mature miRNAs, by enhancing Dicer‐mediated precursor processing and/or loading of miRNAs onto RISCs.^23^, ^24^ Interestingly, enoxacin significantly increased the invasion ability of HTR8 cells (Figure 1F), indicating that enhancing the DICER activity of RNA interference markedly increased the invasion ability of HTR8 cells. Collectively, these results indicate that DICER and DICER‐dependent miRNAs play a key role in regulating the invasion ability of EVT.

### 3.2 DICER knockdown impairs invasion of HTR8/SVneo cells partly through increased miR‐16‐2‐3P

**Figure 2:**
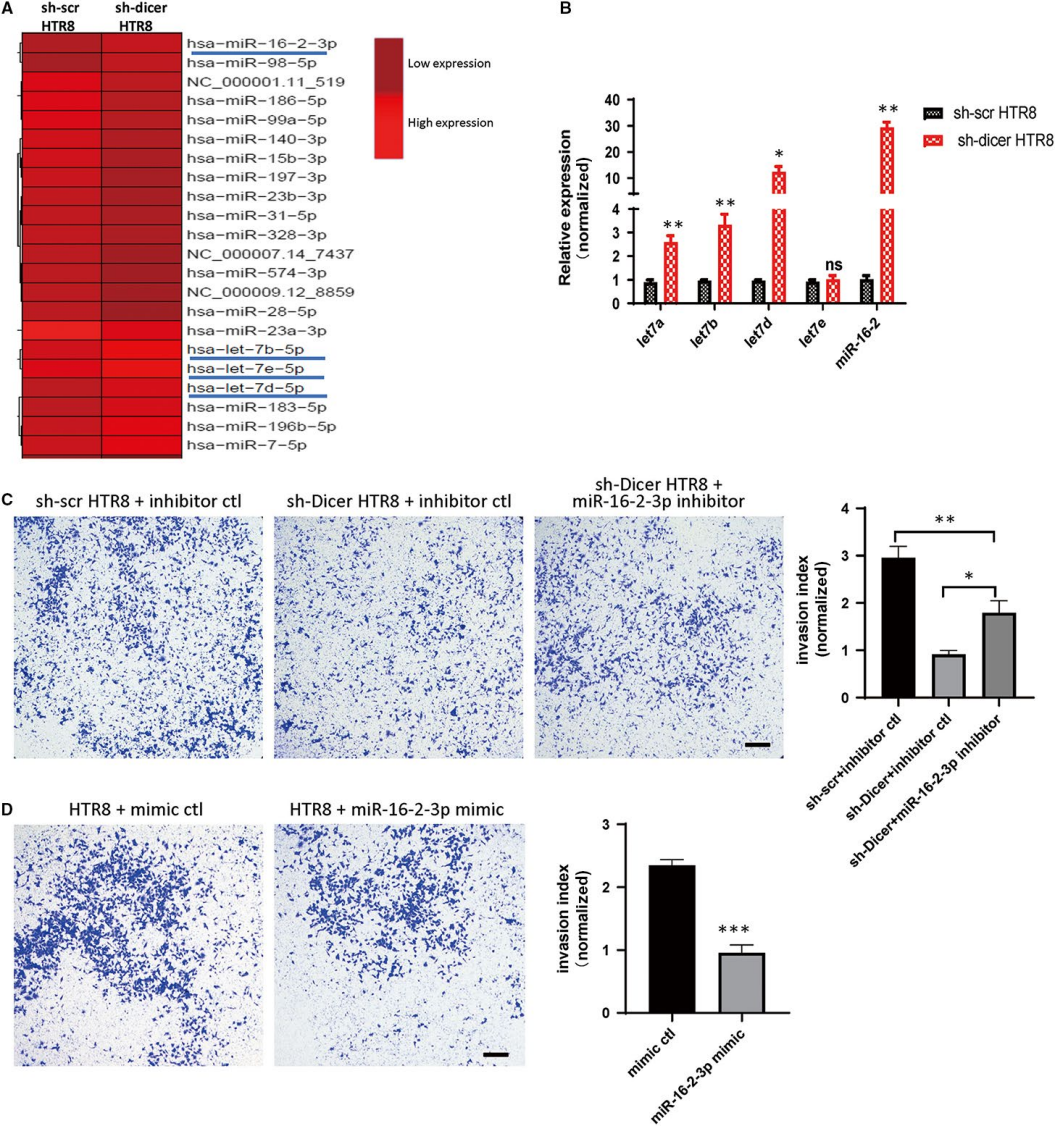
Increased expression of miR‐16‐2‐3p is responsible for the reduced invasion ability of sh‐Dicer HTR8 cells. A, Heat map of miRNAs by miRNA sequence in sh‐scr and sh‐Dicer HTR8 cells. miRNAs of high abundance and significant change are underlined. High relative expression in light red and low relative expression in dark red. B, miRNA levels (including let‐7a, let‐b, let‐7d, let‐7e and miR‐16‐2) relative to U6 determined by qPCR in sh‐scr and sh‐Dicer HTR8 cells. The mean ± SEM is shown. *P < .05, **P < .01, ns, non‐significant (n = 3). C, invasion assay of sh‐scr HTR8 cells transfected with miRNA inhibitor control (inhibitor ctl) and sh‐Dicer HTR8 cells transfected with miRNA inhibitor ctl or miR‐16‐2‐3p inhibitor (left). Scale bar = 200 µm. Quantification of cell number at the outside membrane of transwell insert (right). The mean ± SEM is shown. *P < .05, **P < .01 (n = 3). D, Invasion assay of HTR8 cells transfected with miRNA mimic control (mimic ctl) or miR‐16‐2‐3p mimic (left). Scale bar = 200 µm. Quantification of cell number at the outside membrane of transwell insert (right). The mean ± SEM is shown. *P < .05, ***P < .001 (n = 3)

Since DICER is a key rate‐limiting enzyme in the biogenesis of miRNAs, we assessed how changed miRNAs levels were involved in the reduced invasion ability of sh‐Dicer HTR8. A miRNA sequencing was performed to compare the miRNA expression profile between sh‐Dicer and sh‐scr HTR8 cells. Results showed that the expression of most miRNAs was down‐regulated, as expected, while the expression of several miRNAs with high abundance was significantly up‐regulated, including a few members of let‐7 family and miR‐16‐2 (Figure 2A). Then, real‐time PCR (RT‐PCR) was performed to confirm the expression of several members of the let‐7 family (let‐7a‐5p, let‐7b‐5p, let‐7d‐5p, let‐7e‐5p) and miR‐16‐2‐3p. In agreement with the miRNA sequencing results, miR‐16‐2‐3p and let‐7d‐5p expression increased dramatically upon DICER knockdown (Figure 2B).Considering the significant changes in let‐7d and miRNA‐16‐2 levels and their well‐studied roles in inhibiting invasion of various cells,^25^, ^26^ we further explored whether up‐regulated miR‐16‐2‐3p or let‐7d‐5p could mediate the attenuated invasion ability of sh‐Dicer HTR8 cells. Invasion assay showed that miR‐16‐2‐3p knockdown partly recovered the invasion ability of sh‐Dicer HTR8 cells, the recovery effect of which was better than let‐7d‐5p knockdown (Figure 2C; Figure S2). Moreover, miR‐16‐2‐3p overexpression impaired the invasion ability of sh‐Dicer HTR8 cells (Figure 2D). Together, these results indicate that miR‐16‐2‐3p partly mediate the impaired invasion ability of sh‐Dicer HTR8 cells.

### 3.3 COL1A2 is a bona fide miR‐16‐2 target mediating the invasion of sh‐Dicer HTR8 cells

**Figure 3:**
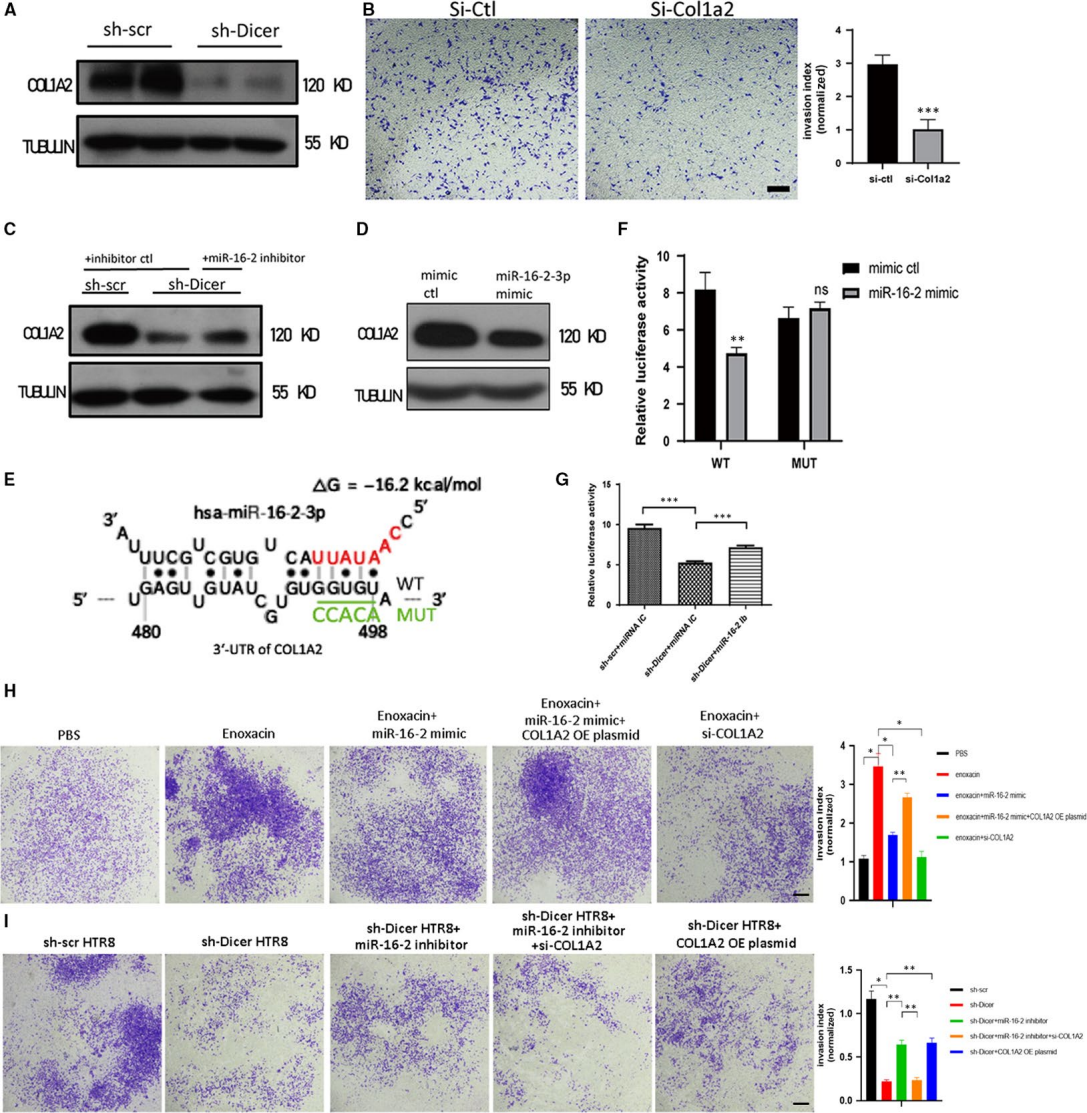
COL1A2 is a target of miR‐16‐2‐3p and mediates the impaired invasion of sh‐Dicer HTR8 cells. A, Representative immunoblot of COL1A2 in sh‐scr and sh‐Dicer HTR8 cells. TUBULIN was blotted as loading control. B, Invasion assay of HTR8 cells transfected with si‐ctl or si‐Col1a2 RNA (left). Scale bar = 200 µm. Quantification of cell number at the outside membrane of transwell insert (right). The mean ± SEM is shown. ***P < .001 (n = 3). C, Representative immunoblot of COL1A2 in sh‐scr HTR8 cells transfected with miRNA inhibitor ctl and sh‐Dicer HTR8 cells transfected with inhibitor ctl or miR‐16‐2‐3p inhibitor. TUBULIN was blotted as loading control. D, Representative immunoblot of COL1A2 in HTR8 cells transfected with miRNA mimic ctl or miR‐16‐2‐3p mimic. TUBULIN was blotted as loading control. E, Schematic diagram shows one predicted binding site of miR‐16‐2‐3p on the wild‐type 3′‐UTR of COL1A2 (WT) where red bases in miR‐16‐2‐3p represent its seed region. Five bases underlined by green line in WT are changed into five other bases shown in green colour and referred as to MUT. F, Relative luciferase activity (firefly luminescence/Renilla luminescence) of HTR8 cells 48 h after transfection of psiCHECK‐2 vector with the COL1A2 WT or MUT 3′‐UTR insert. miR‐16‐2 mimic or mimic ctl is transfected into HTR8 cells 24 h before luciferase activity is measured. The mean ± SEM is shown. **P < .001, ns, non‐significant (n = 3). G, Relative luciferase activity (firefly luminescence/Renilla luminescence) of sh‐Dicer or sh‐scr HTR8 cells 48 h after psiCHECK‐2 vector with the COL1A2 WT 3′‐UTR insert is transfected. miR‐16‐2 inhibitor or inhibitor control is transfected into sh‐Dicer HTR8 cells or sh‐scr HTR8 cells 24 h before luciferase activity is measured. The mean ± SEM is shown. ***P < .001 (n = 3). H, Invasion assay of HTR8 cells treated with enoxacin or transfected with miR‐16‐2‐3p mimic or si‐Col1a2 RNA or COL1A2 overexpression plasmid. Scale bar = 200 µm. Quantification of cell number at the outside membrane of transwell insert (right). The mean ± SEM is shown. *P < .05, **P < .01 (n = 3). I, Invasion assay of sh‐scr HTR8 cells and sh‐Dicer HTR8 cells transfected with miR‐16‐2‐3p inhibitor or si‐Col1a2 RNA or COL1A2 overexpression plasmid. Scale bar = 200 µm. Quantification of cell number at the outside membrane of transwell insert (right). The mean ± SEM is shown. *P < .05, **P < .01 (n = 3)

To identify the direct downstream target through which miR‐16‐2‐3p regulates invasion of HTR8 cells, three candidates were picked up based on previous reports and bioinformatics analyses. COL1A2, a protein highly expressed in placenta and positively associated with various cancer metastasis,^27^, ^28^, ^29^ is a predicted target of miR‐16‐2‐3p in an incomplete complimentary pairing manner, as shown by the STarMir module of Sfold software (http://sfold.wadsworth.org/cgi-bin/index.pl). In addition, MMP2 and MMP9, members of matrix metalloproteinases (MMPs), have been reported to promote invasion and to be down‐regulated by miR‐16‐2.^30^ Therefore, the expression level of these three proteins was detected in sh‐Dicer and sh‐scr HTR8 cells. Surprisingly, the protein level of COL1A2 was significantly decreased in sh‐Dicer HTR8 cells, but not the protein level of MMP2 and MMP9 (Figure 3A; Figure S3A). To test whether the decrease of the COL1A2 protein mediated the reduced invasion ability of sh‐Dicer HTR8 cells, the invasion ability of HTR8 cells transfected with COL1A2 siRNA (si‐COL1A2) was determined. Consistently, the invasion ability of si‐COL1A2 HTR8 cells decreased significantly (Figure 3B; Figure S3B‐C). Then, we explored whether COL1A2 is target of miR‐16‐2‐3p. Immunoblot results showed that the protein level of COL1A2 could be partly recovered in sh‐Dicer HTR8 cells treated with miR‐16‐2‐3p inhibitor and was decreased in HTR8 cells transfected with a miR‐16‐2‐3p mimic (Figure 3C‐D). Next, the 3′‐UTR of COL1A2 was cloned into a psiCHENCK2 plasmid (WT) to detect whether COL1A2 was a target of miR‐16‐2 by the luciferase assay. Meanwhile, a mutant plasmid (MUT) was constructed, where a few base pairs were changed in the miR‐16‐2‐3p predicted binding site (Figure 3E). Consistently, the luciferase assay showed that miR‐16‐2‐3p mimic significantly reduced the relative luciferase activity of the WT plasmid, but not the MUT plasmid in HTR8 cells (Figure 3F). In addition, miR‐16‐2‐3p knockdown could partly recover the relative luciferase activity of WT plasmid in sh‐Dicer HTR8 cells (Figure 3G). To test the effects of DICER‐miR‐16‐2‐3p‐COL1A2 axis on invasion of HTR8 cells, a series of invasion assays were performed. Results showed the increased invasion ability of HTR8 cells by enoxacin could be offset by miR‐16‐2‐3p mimic and si‐COL1A2 RNA, respectively. And overexpression of COLA2 successfully recovered the invasion ability of HTR8 cells treated by enoxacin and miR‐16‐2‐3p mimic simultaneously (Figure 3H; Figure S3D). Moreover, overexpression of COL1A2 or miR‐16‐2‐3p inhibitor could partly recover the invasion ability of sh‐Dicer HTR8 cells, respectively. And si‐COL1A2 RNA reduced the invasion ability of sh‐Dicer HTR8 cells recovered by miR‐16‐2‐3p inhibitor (Figure 3I). In conclusion, the decreased protein level of COL1A2, targeted by miR‐16‐2‐3p, is at least partially responsible, for the decreased invasion ability of sh‐Dicer HTR8 cells.

### 3.4 DICER knockdown in HTR8 cells impairs the pro‐angiogenetic effect of microvesicles on HUVECs

**Figure 4:**
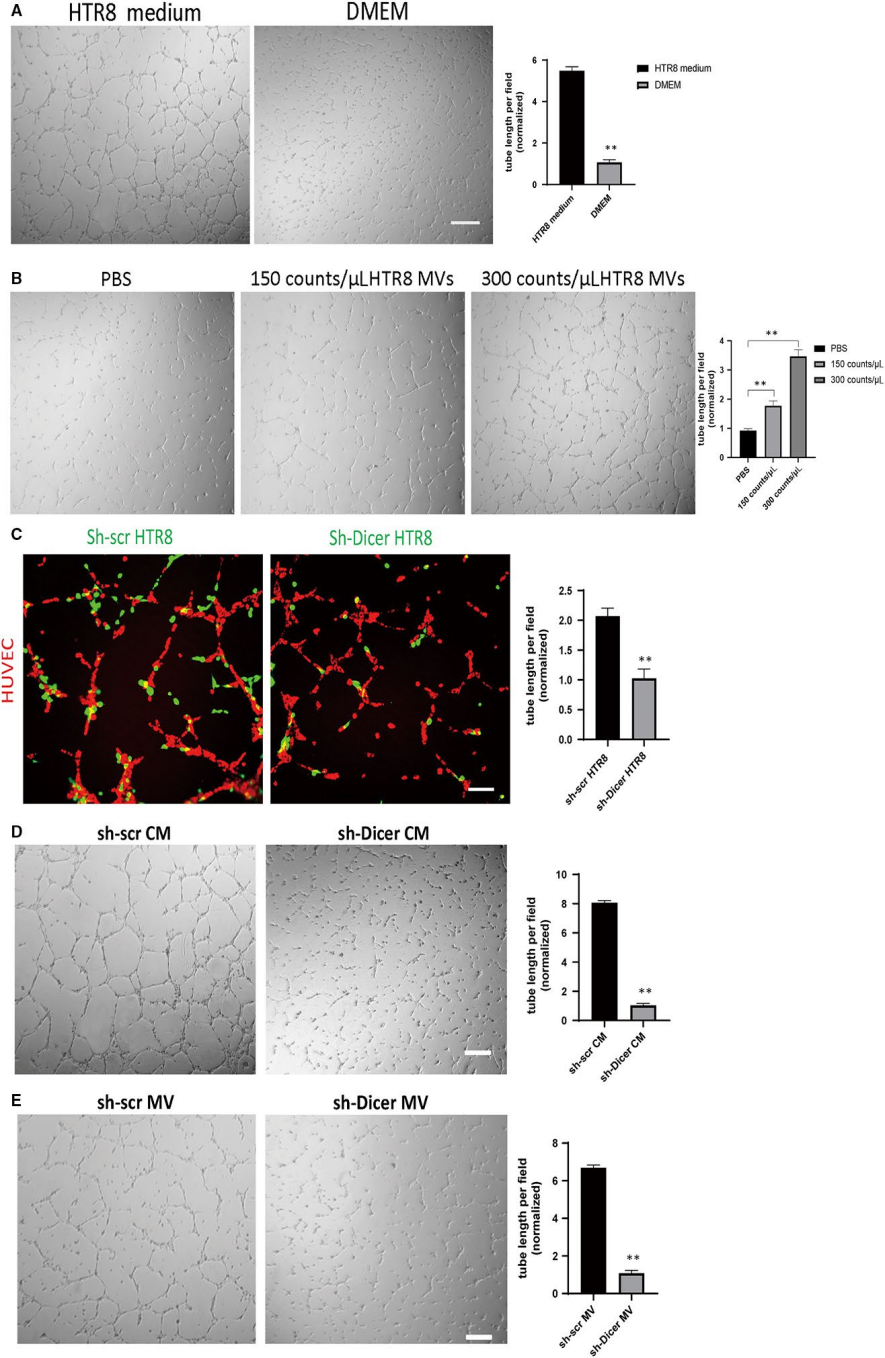
DICER knockdown in HTR8 cells impairs the pro‐angiogenetic effect of microvesicles on HUVECs. A, Tube formation assay of HUVECs treated with HTR8 CM or DMEM. Scale bar = 200 µm. Quantification of tube length formed by HUVECs (right). The mean ± SEM is shown. **P < .01, ns, non‐significant (n = 3). B, Tube formation assay of HUVECs treated with different concentration of HTR8 MVs. Scale bar = 200 µm. Quantification of tube length formed by HUVECs (right). The mean ± SEM is shown. **P < .01, ns, non‐significant (n = 3). C, Tube formation assay of red cell tracker stained HUVECs cocultured with green cell tracker stained sh‐scr or sh‐Dicer HTR8 cells at the ratio of 2:1 (left). Scale bar = 130 µm. Quantification of tube length formed by HUVECs (right). The mean ± SEM is shown. **P < .01, (n = 3). D, Tube formation assay of HUVECs treated with sh‐scr or sh‐Dicer CM supplemented with 2% FBS (left). Scale bar = 200 µm. Quantification of tube length formed by HUVECs (right). The mean ± SEM is shown. **P < .01, (n = 3). E, Tube formation assay of HUVECs treated with purified sh‐scr or sh‐Dicer MVs resuspended in DMEM supplemented with 2% FBS (left). Scale bar = 200 µm. Quantification of tube length formed by HUVECs (right). The mean ± SEM is shown. **P < .01, (n = 3)

It has been reported that EVT can adhere to endothelial cells and promote angiogenesis, which is mediated by cell adhesion protein N‐cadherin expressed on EVT.^31^ However, the mechanisms of how EVT interact with endothelial cells during spiral artery remodelling are still unclear. Here, the tube formation ability of HUVECs was observed by a tube formation assay, following treatment with conditioned medium (CM) collected from HTR8 cells (HTR8‐CM). The results showed that HTR8‐CM significantly promoted tube formation by HUVECs as compared to the DMEM control medium, indicating that HTR8 cells could promote angiogenesis indirectly (Figure 4A). To identify whether the EVs contained in the HTR8‐CM mediated its pro‐angiogenic effect, we firstly explored the types of EVs in the HTR8‐CM by nanoparticle tracking analysis (NTA). The results showed that most EVs in HTR8‐CM were MVs with 500nm of diameter, while only a minority of EVs were exosomes with a diameter of <100 nm (Figure S4A). Then, a coculture model of green cell tracker labelled HTR8 cells and HUVECs was established to determine whether MVs derived from the membranes of HTR8 cells (HTR8 MVs) could be absorbed by HUVECs. The results showed that some of the HUVECs on the bottom attain green fluorescence after 24 hours of coculture (Figure S4B‐C). To test whether these HTR8 MVs mediated the pro‐angiogenic effect of HTR8 CM, HUVECs were then treated with a range of concentrations of HTR8 MVs. Consistently, HTR8 MVs showed pro‐angiogenetic effect on HUVECs (Figure 4B). Overall, EVs secreted from HTR8 cells, which were mostly MVs, could promote angiogenesis of HUVECs.

Next, we further explored whether this interaction mediated by EVT‐derived MVs could be affected after DICER knockdown in EVT. Interestingly, the tube formation ability of HUVECs cocultured with sh‐scr HTR8 cells was significantly better than those cocultured with sh‐Dicer HTR8 cells (Figure 4C). To explore whether sh‐dicer HTR8 cells mediated this different effect of angiogenesis in an indirect way, we firstly compared the tube formation ability of HUVECs treated with CM collected from sh‐scr or sh‐dicer HTR8 cells separately (named as sh‐scr CM and sh‐Dicer CM, respectively). Consistently, HUVECs treated with sh‐scr CM had significantly better tube formation ability, compared to those treated with sh‐Dicer CM (Figure 4D). We then treated HUVECs with MVs purified from sh‐scr or sh‐Dicer CM. The results showed that the effect of sh‐scr MVs on tube formation by HUVECs was better than sh‐Dicer MVs (Figure 4E). In conclusion, DICER protein is necessary for the crosstalk between HTR8 cells and HUVECs mediated by HTR8 cell‐derived MVs.

### 3.5 Reduced DICER protein and increased miR‐16‐2‐3P and let‐7d‐5p in sh‐Dicer MVs contribute to an anti‐angiogenic effect in sh‐Dicer CM

**Figure 5:**
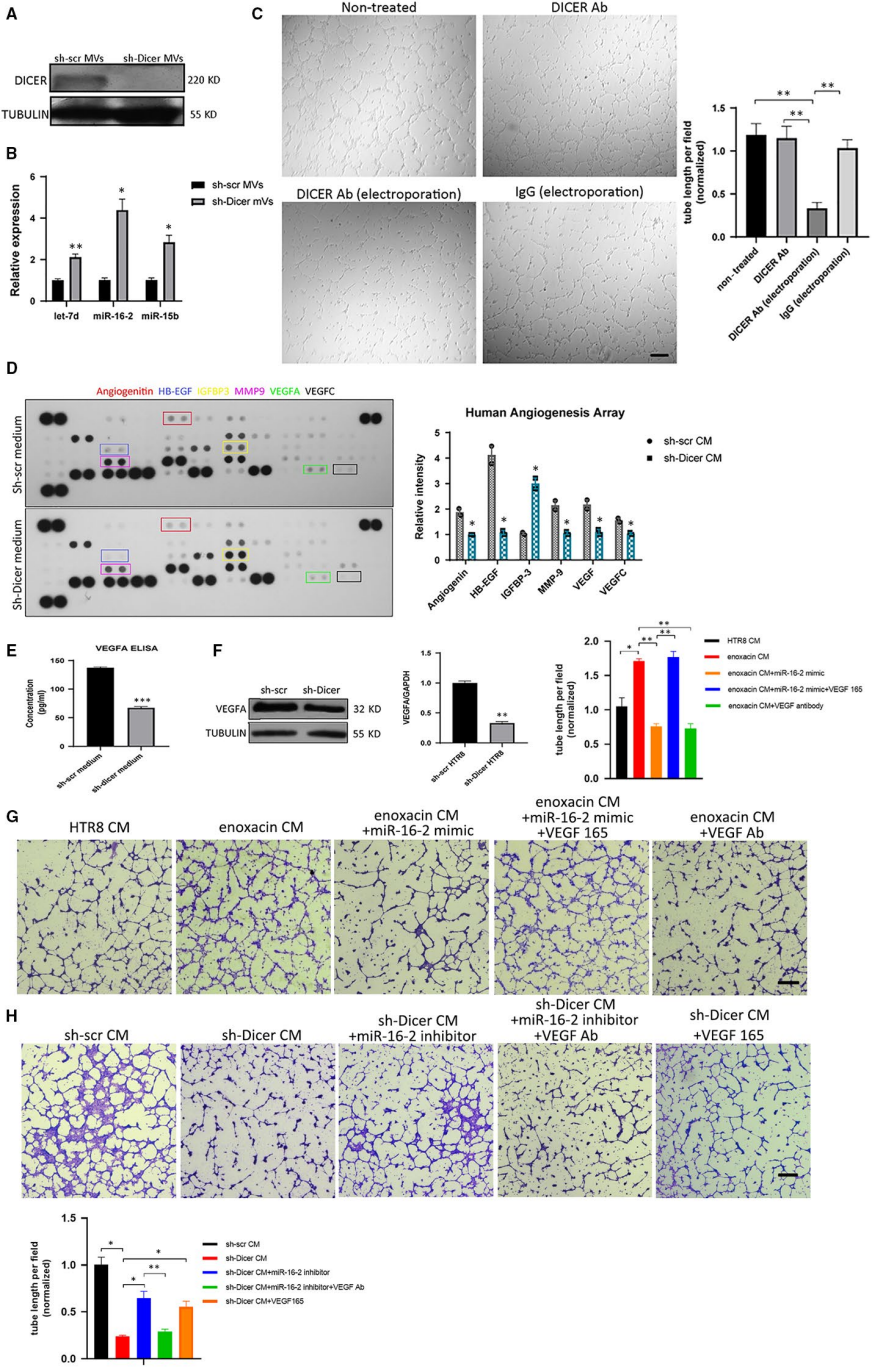
Reduced VEGFA, DICER protein and increased miR‐16‐2‐3P and let‐7d‐5p were found in the sh‐Dicer CM and sh‐Dicer MVs, respectively. A, Representative immunoblotting of DICER in sh‐scr and sh‐Dicer MVs. TUBULIN is used as loading control. B, miRNAs levels (let‐7d‐5p, miR‐16‐2‐3p and miR‐15b) relative to U6 determined by qPCR in sh‐scr and sh‐Dicer HTR8 MVs). The mean ± SEM is shown. *P < .05, **P < .01, (n = 3). C, Tube formation assay of HUVECs treated with HTR8 MVs suspended in DMEM with different treatment (A: HTR8 MVs only; B: HTR8 MVs supplemented by DICER Ab; C: HTR8 MVs electroporated with DICER Ab; and D: HTR8 MVs electroporated with IgG). Scale bar = 300 µm. Quantification of tube length formed by HUVECs (right). The mean ± SEM is shown. **P < .01, (n = 3). D, Human angiogenesis array of sh‐scr and sh‐Dicer CM. Some angiogenesis‐related factors with significant change of expression are highlighted (right). Normalized relative intensity of black dot of angiogenic factors (left). The mean ± SEM is shown. **P < .01, (n = 2). E, Quantification of VEGFA protein concentration in sh‐scr and sh‐Dicer CM by ELISA. The mean ± SEM is shown. ***P < .001, (n = 3). F, VEGFA protein level (left) and mRNA level (right) in sh‐scr and sh‐Dicer HTR8 cells by immunoblotting and RT‐PCR, respectively. The mean ± SEM is shown. ** *P* < .01, (n = 3). G, Tube formation assay of HUVECs treated with CM derived from different sources (HTR8 cells; HTR8 cells treated with enoxacin and/or VEGF Antibody, VEGF165 and/or transfected with miR‐16‐2‐3p mimic). Scale bar = 200 µm. Quantification of tube length formed by HUVECs (upper right). The mean ± SEM is shown. *P < .05, **P < .01, (n = 3). H, Tube formation assay of HUVECs treated with sh‐scr CM or sh‐Dicer CM where sh‐Dicer cells were transfected miR‐16‐2‐3p and/or sh‐Dicer CM was treated with VEGF antibody or VEGF165. Scale bar = 200 µm. Quantification of tube length formed by HUVECs (bottom left). The mean ± SEM is shown. *P < .05, **P < .01, (n = 3)

To test whether the dysregulated levels of DICER protein and miRNAs in sh‐Dicer HTR8 cells were transmitted to MVs, the levels of DICER protein and three anti‐angiogenic miRNAs (including let‐7d, miR‐16‐2 and miR‐15b) were detected in purified MVs by immunoblotting and RT‐PCR, respectively.^32^, ^33^ Consistently, the protein level of DICER was reduced and these three miRNAs levels were up‐regulated in the sh‐Dicer MVs (Figure 5A‐B). To explore whether the decreased protein level of DICER in sh‐Dicer MVs has an impact on the tube formation ability of HUVECs, we blocked the function of the DICER protein in sh‐scr MVs by electroporation of a DICER antibody (DICER Ab), to mirror the deficiency of DICER protein in sh‐Dicer MVs. The tube formation assay showed that sh‐scr MVs electroporated with DICER Ab had a significantly reduced pro‐angiogenic effect on HUVECs, compared to those electroporated with IgG (Figure 5C). Moreover, the inhibitory effect of sh‐Dicer CM on angiogenesis was partly recovered when sh‐Dicer HR8 cells were transfected with miR‐16‐2‐3p inhibitor, indicating that increased expression of miR‐16‐2‐3p in CM played an inhibitory role in angiogenesis (Figure S5A). In conclusion, the increased levels of anti‐angiogenic miRNAs and the decreased protein level of DICER in MVs were associated with an impaired angiogenic ability of sh‐Dicer MVs. Therefore, there was dysregulated expression of miRNAs and DICER protein in the sh‐Dicer MVs, and the decreased protein level of DICER in MVs was linked to an impaired angiogenic ability of sh‐Dicer MVs.

To figure out whether the DICER knockdown in HTR8 cells affects the secretion profile of angiogenic factors in sh‐Dicer CM, a human angiogenesis array was performed. The results showed that the levels of several angiogenic factors were markedly higher in sh‐scr CM (including angiopoietin, HB‐EGF, MMP9, VEGFA and VEGFC) and that the level of IGFBP3, one anti‐angiogenic factor, was significantly decreased as compared to sh‐Dicer CM (Figure 5D).^34^, ^35^, ^36^, ^37^ The decreased expression level of VEGFA in CM was further selectively identified by ELISA (Figure 5E). Consistently, both the mRNA and the protein level of VEGFA were decreased in sh‐Dicer HTR8 cells (Figure 5F), indicating that the decreased protein level of VEGFA in sh‐Dicer CM resulted from a decreased protein level of VEGFA in sh‐Dicer HTR8 cells. In addition, adding moderate amount of VEGF165 cytokine in sh‐Dicer CM could significantly recover the tube formation ability of HUVECs (Figure S5B). To test the effects of DICER‐miR‐16‐2‐3p‐VEGFA axis on angiogenesis, a series of tube formation assays were performed. Results showed increased pro‐angiogenic effect of CM derived from enoxacin‐treated HTR8 cells (enoxacin CM), compared with that from PBS‐treated HTR8 cells (HTR8 CM). This pro‐angiogenic effect of enoxacin could be offset by miR‐16‐2‐3p mimic and VEGF antibody, respectively. And VEGF165 cytokine successfully recovered the pro‐angiogenic effect of enoxacin CM inhibited by miR‐16‐2‐3p mimic (Figure 5G). Moreover, VEGF165 cytokine and miR‐16‐2‐3p inhibitor could partly recover the anti‐angiogenic effect of sh‐Dicer CM, respectively. And VEGF antibody reduced the pro‐angiogenic effect of sh‐Dicer CM recovered by miR‐16‐2‐3p inhibitor (Figure 5H). Altogether, these results show that this DICER‐miR‐16‐2‐3p‐VEGFA axis plays a co‐ordinating role in regulating angiogenesis, where a reduced DICER protein level in sh‐Dicer MVs leads to reduced angiogenesis and that, following DICER knockdown, increased anti‐angiogenic miRNAs and aberrant expression of angiogenic factors lead to an anti‐angiogenic effect in sh‐Dicer CM.

### 3.6 The protein level of DICER is decreased in the placenta of PE patients

**Figure 6:**
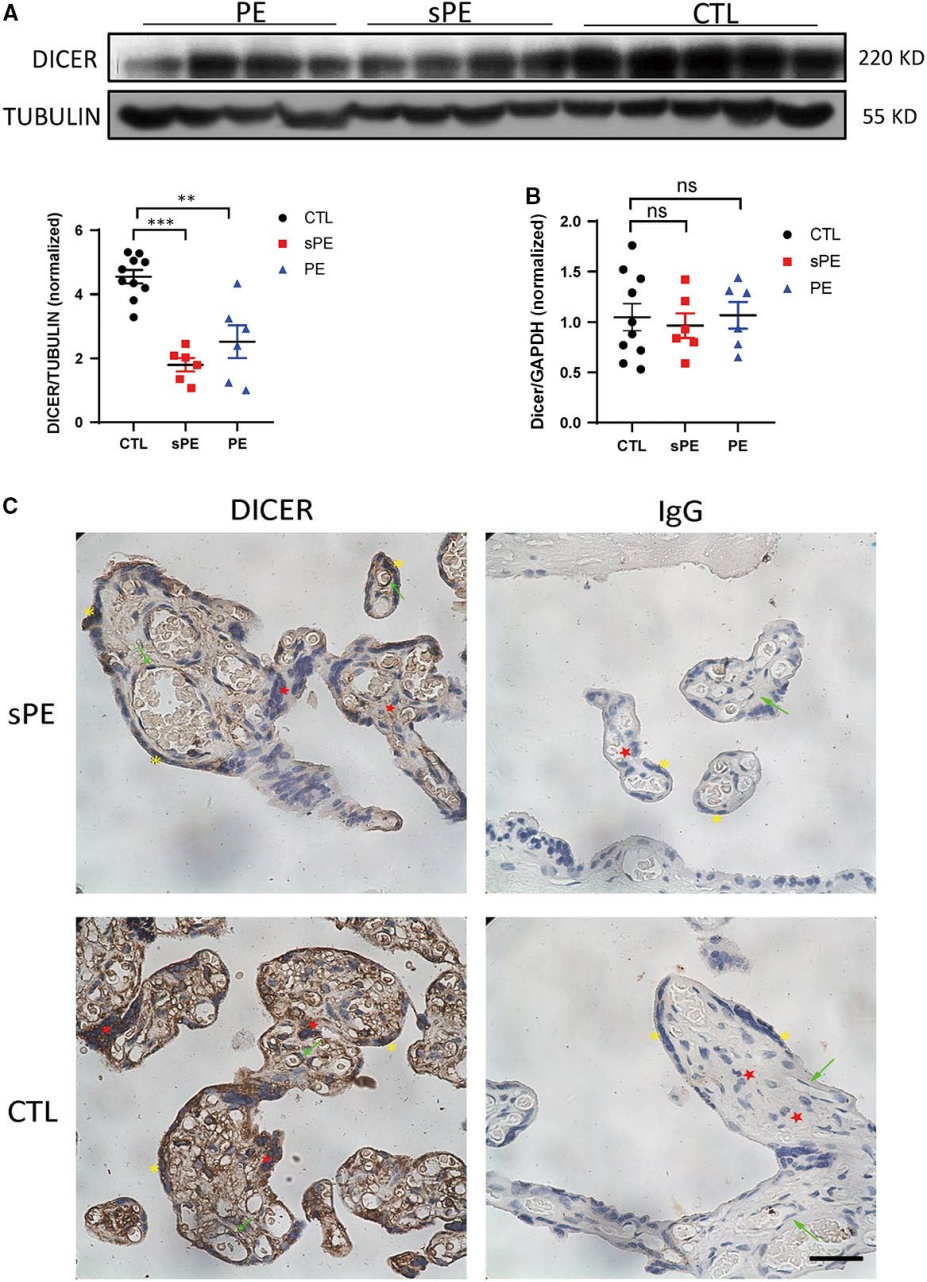
Decreased expression of DICER protein in PE placenta. A, Representative immunoblot of DICER in placenta from PE, sPE patients and control normotensive pregnant women (CTL) at delivery. TUBULIN was blotted as loading control (top). Intensity ratio of DICER to TUBULIN (lower left) in CTL group, sPE group and PE group. The mean ± SEM is shown. **P < .01 and ***P < .001 (n = 10 for CTL, n = 6 for PE and n = 6 for sPE). B, DICER mRNA levels relative to GAPDH in placenta from PE, sPE patients and normotensive pregnant women at delivery determined by qPCR. The mean ± SEM is shown. ns, non‐significant, (n = 10 for CTL, n = 6 for PE and n = 6 for sPE). C, DICER immunohistochemistry‐stained placental sections from sPE patients and normotensive pregnant women where red star labels CTB, yellow asterisk labels STB and green arrow labels endothelial cells. Scale bar = 150 µm

Since DICER has been proved to be necessary for maintaining normal function of EVT, including invasion and communication with endothelial cells, we further explored whether DICER is involved in the pathogenesis of pregnant complications attributed to placental insufficiency, such as PE and FGR. To do this, we compared DICER expression in placentas from normal pregnancies and from pregnancies with complications. PE is a common pregnancy complication and is characterized by hypertension, proteinuria and systemic endothelial dysfunction.^38^, ^39^ Specifically, according to the clinical manifestations, PE can be divided into PE and sPE. Surprisingly, we found that DICER protein was significantly decreased in PE placenta (both in PE and sPE) by Western blot (Figure 6A). However, the mRNA level of DICER did not decrease in PE or sPE placenta, which is consistent with a previous study (Figure 6B).^13^ Importantly, IHC analysis of placenta showed that DICER protein was reduced both in the CTB and STB (Figure 6C). These data imply that DICER might play an important role in placentation and possibly be required to prevent pathogenesis of PE.

**Figure 7:**
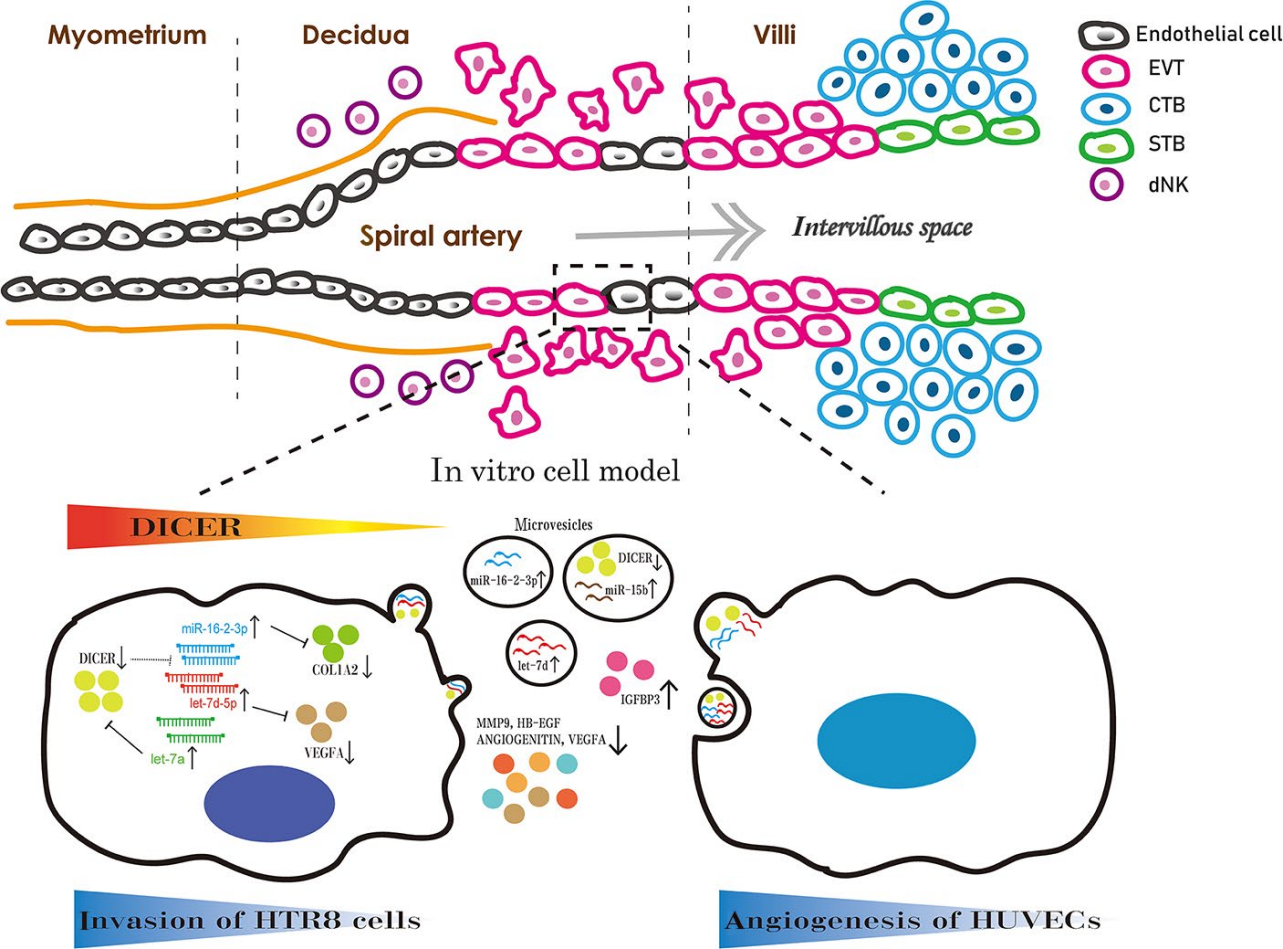
Schematic diagram shows the proposed mechanism of DICER mediating the invasion ability of HTR8 cells and the crosstalk between HTR8 cells and HUVECs during spiral artery remodelling in an in vitro model. DICER plays an important role in balancing the expression of miR‐16‐2 and let‐7 family, and there seems to be a positive feedback between DICER and let‐7 family where DICER inhibits the transcription of MIRLET7A1 gene and DICER is a target of let‐7a (49). Increased level of miR‐16‐2‐3p leads to decreased invasion ability and VEGFA secretion by targeting COL1A2 and VEGFA, respectively, in HTR8 cells following DICER knockdown. Silencing of DICER also creates an anti‐angiogenic bias in the extracellular microenvironment of HTR8 cells with dysregulated angiogenic factors (up‐regulated IGFBP3, down‐regulated VEGFA, HB‐EGF, MMP9 and ANGIOGENITIN), and increased miRNAs (miR‐16‐2, miR‐15b and let‐7d) and decreased DICER protein in HTR8 cell‐derived MVs, leading to impaired angiogenesis of HUVECs

Figure 7 presents the proposed mechanism of how DICER mediates the invasion ability of HTR8 cells and the crosstalk between HTR8 cells and HUVECs, during spiral artery remodelling.

## 4 DISCUSSION

In the present study, we provide the first mechanistic insight of how DICER controls the invasion ability of HTR8 cells and illustrate the mechanisms in which DICER mediates the crosstalk between trophoblasts and endothelial cells via DICER‐containing microvesicles. Employing our DICER knockdown cell model, we found that DICER deficiency remarkably impaired the invasion ability of both primary EVT and the HTR8 cell line, indicating that DICER is necessary for their invasion ability. Given that the processing of miRNAs is inhibited in DICER knocked‐down trophoblasts, it is implied that the balance of miRNAs levels regulated by DICER is critical for maintaining the invasive behaviour of trophoblasts.

Small RNA sequencing was then employed to identify abnormal expression of miRNAs in sh‐Dicer HTR8 cells. Among the dysregulated miRNAs, miR‐16‐2‐3p and let‐7d‐5p were proved to be the two miRNAs with the most significantly altered expressions. Considering that the processing of miRNAs was limited in sh‐Dicer HTR8 cells, it was hypothesized that increased levels of miRNAs resulted from activation of transcription. Then, we want to test whether this was the case for the up‐regulated miR‐16‐2. Pri‐miR‐16‐2 derives from chromosome 3q25, which is intronic to SMC4, a gene encoding for structural maintenance of chromosomes protein 4 (SMC4).^40^ Since miR‐16‐2 is co‐transcribed with SMC4, as reported for most intronic miRNAs, we measured the expression level of SMC4 by RT‐PCR, as an indirect indication of the transcriptional activity of miR‐16‐2. Consistently, the expression of SMC4 mRNA was up‐regulated. In addition, the expression of pre‐miR‐16‐2 was also significantly increased. Moreover, the expression of miR‐15b, which is also intronic to the SMC4 gene, was increased as well (Figure S6). These results suggest that the overexpression of miRNAs may result from transcriptional activation of the miRNA genes in sh‐Dicer HTR8 cells, though the specific transcription factor was not identified. Thus, it is inferred that the specific interplay between DICER and miRNAs differs in varied intracellular environments, given the fact that the correlation between DICER and let‐7 seems to be inconsistent in different cell types where the expression of let‐7 can be positively or negatively associated with DICER protein levels.^41^, ^42^, ^43^

After demonstrating that up‐regulated miR‐16‐2‐3p mainly contributes to decreased invasion of DICER knockdown HTR8 cells, we further explore the target protein of miR‐16‐2‐3p that regulates invasion. Interestingly, the protein expression of COL1A2 is dramatically down‐regulated in sh‐Dicer HTR8 cells, which can further lead to an impaired invasion ability. Moreover, the COL1A2 protein is a bona fide target of the up‐regulated miR‐16‐2‐3p, as demonstrated by the luciferase assay, suggesting the DICER‐miR‐16‐2‐COL1A2 mediated pathway in regulating invasion of EVT.

One novel finding of the current study is the critical role of DICER involved in the crosstalk between HTR8 cells and HUVECs. Firstly, our results show that HTR8 MVs have a pro‐angiogenic effect on tube formation of HUVECs. Secondly, sh‐Dicer HTR8 cells impair the tube formation ability of HUVECs both in a direct way and in an MV‐mediated way. Although how sh‐Dicer HTR8 cells directly reduce the tube formation of HUVECs is not studied yet, the indirect role of sh‐Dicer HTR8 cells in angiogenesis can be mediated by the sh‐Dicer MVs in the CM. Finally, the reduced expression of DICER in HTR8 cells can be transmitted to sh‐Dicer MVs and affect angiogenesis negatively, consistent with previous reports on the role of DICER in angiogenesis.^44^, ^45^ Furthermore, the increased expression of the anti‐angiogenic miRNAs in sh‐Dicer HTR8 cells is also transmitted to sh‐Dicer MVs, including let‐7d, miR‐16‐2 and miR‐15b. Moreover, the expression of angiogenesis‐associated factors was dysregulated in sh‐Dicer CM, where several angiogenic factors were significantly down‐regulated and one anti‐angiogenic factor was remarkably up‐regulated, compared to sh‐scr CM. Among these dysregulated angiogenic factors, the expression of VEGFA is noteworthy, as the decreased protein expression of VEGFA might result from up‐regulated miR‐16‐2 and let‐7a, both of which could target VEGFA.^46^, ^47^ Together, this evidence supports that DICER knockdown in HTR8 cells is adverse to tube formation of HUVECs, which is attributed to reduced DICER expression, increased anti‐angiogenic miRNAs in sh‐Dicer MVs and dysregulated angiogenic factors in sh‐Dicer CM.

Based on this evidence showing the important roles of DICER in EVT, we further explored whether the expression of the DICER protein is implicated in the pathogenesis of pregnant complications. Here, we found that there is decreased expression of the DICER protein in the PE placenta, especially in the sPE placenta. However, the expression of DICER in microvesicles or serum/plasma before the onset of PE should be further tested to identify the involving role of DICER in the generation of PE. An important question is how the expression of DICER is regulated during the pathogenesis of preeclampsia. Given that DICER is a sensitive stress response protein, which can be down‐regulated by hypoxia‐induced HIF1**α**, reactive oxygen species and type 1 interferons,^42^, ^48^ it could be inferred that decreased expression of DICER in PE placenta might result from the chronic hypoxic, or inflammatory placental environment in PE patients. Hence, it is also of great importance to study how DICER is regulated by hypoxia and inflammatory cytokines in trophoblasts, the two main causes for PE, to further clarify the mechanisms of how DICER is down‐regulated in the pathogenesis of PE. In addition, an animal model of PE could be established to adequately elucidate the effect of decreased DICER expression on the pathogenesis of PE.

In conclusion, our study demonstrated that a DICER‐miR‐16‐2‐COL1A2 mediated pathway in regulating invasion of EVT. Additionally, it demonstrated that DICER mediated the crosstalk between EVT and endothelial cells by interfering with the balance of angiogenic and anti‐angiogenic factors in MVs and CM secreted from EVT.

## CONFLICT OF INTEREST

The authors declare no conflicts of interest.

## AUTHOR CONTRIBUTIONS

L Tang and W Xu designed experiments and L Tang wrote the manuscript revised by W Xu; L Tang, M Yang and X Li performed the experiments; L Tang and M Yang analysed the data; and L Qin, G He and X Liu collected the patient samples and analysed the data. All authors reviewed the results and approved the final version of the manuscript.
