# Supplementary figures and images for: Deficiency of DICER reduces the invasion ability of trophoblasts and impairs the pro‐angiogenic effect of trophoblast‐derived microvesicles
Source: J Cell Mol Med. 2020 Mar 21;24(9):4915–30. doi: 10.1111/jcmm.14917 (PMC7205818; doi:10.1111/jcmm.14917)

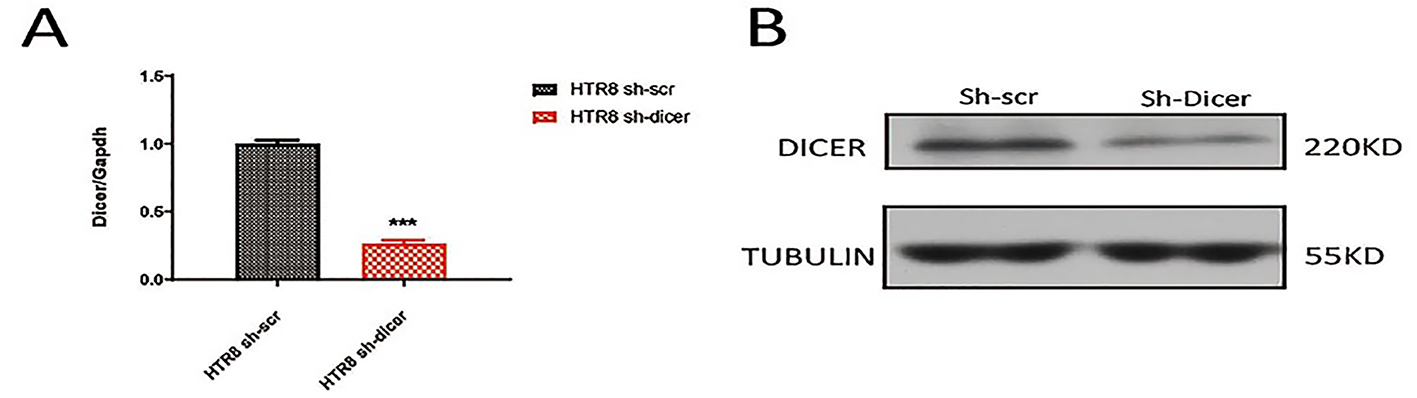

Supplement: Supplementary file 1 [file JCMM-24-4915-s001.tif]

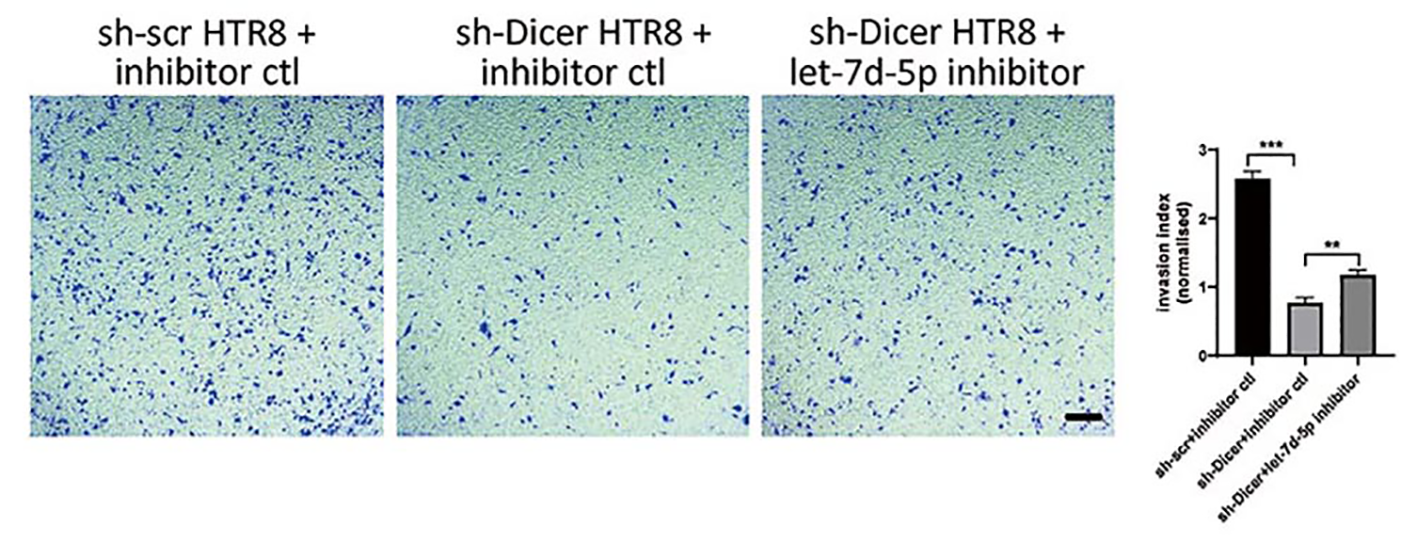

Supplement: Supplementary file 2 [file JCMM-24-4915-s002.tif]

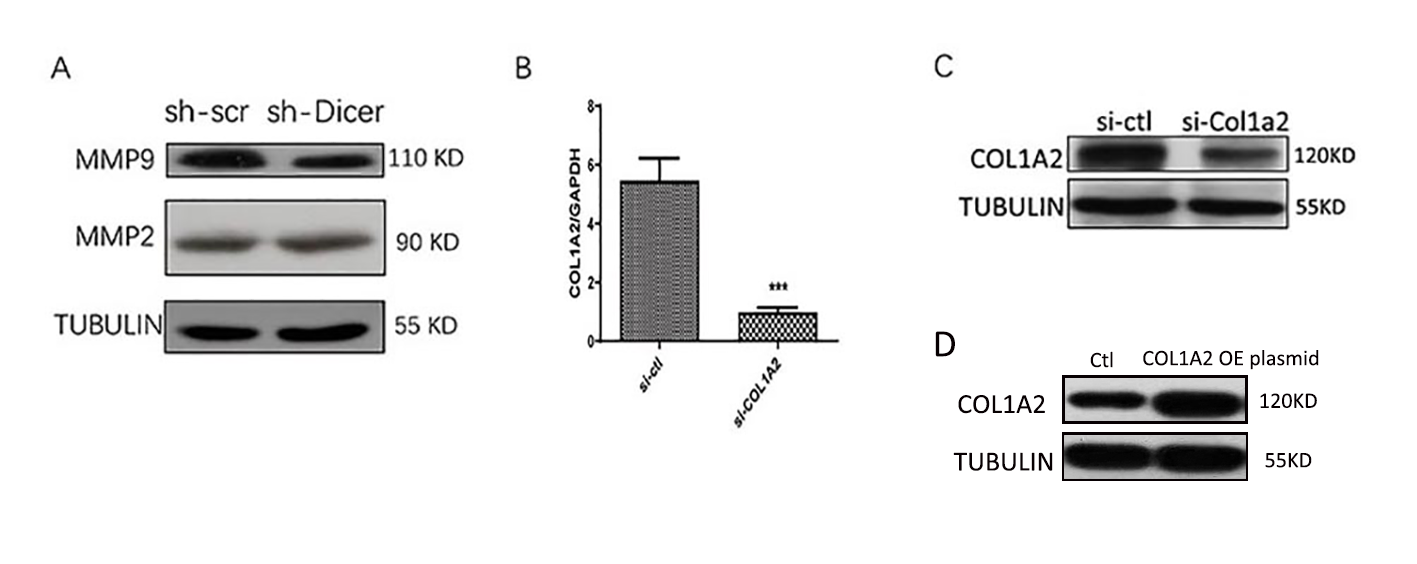

Supplement: Supplementary file 3 [file JCMM-24-4915-s003.tif]

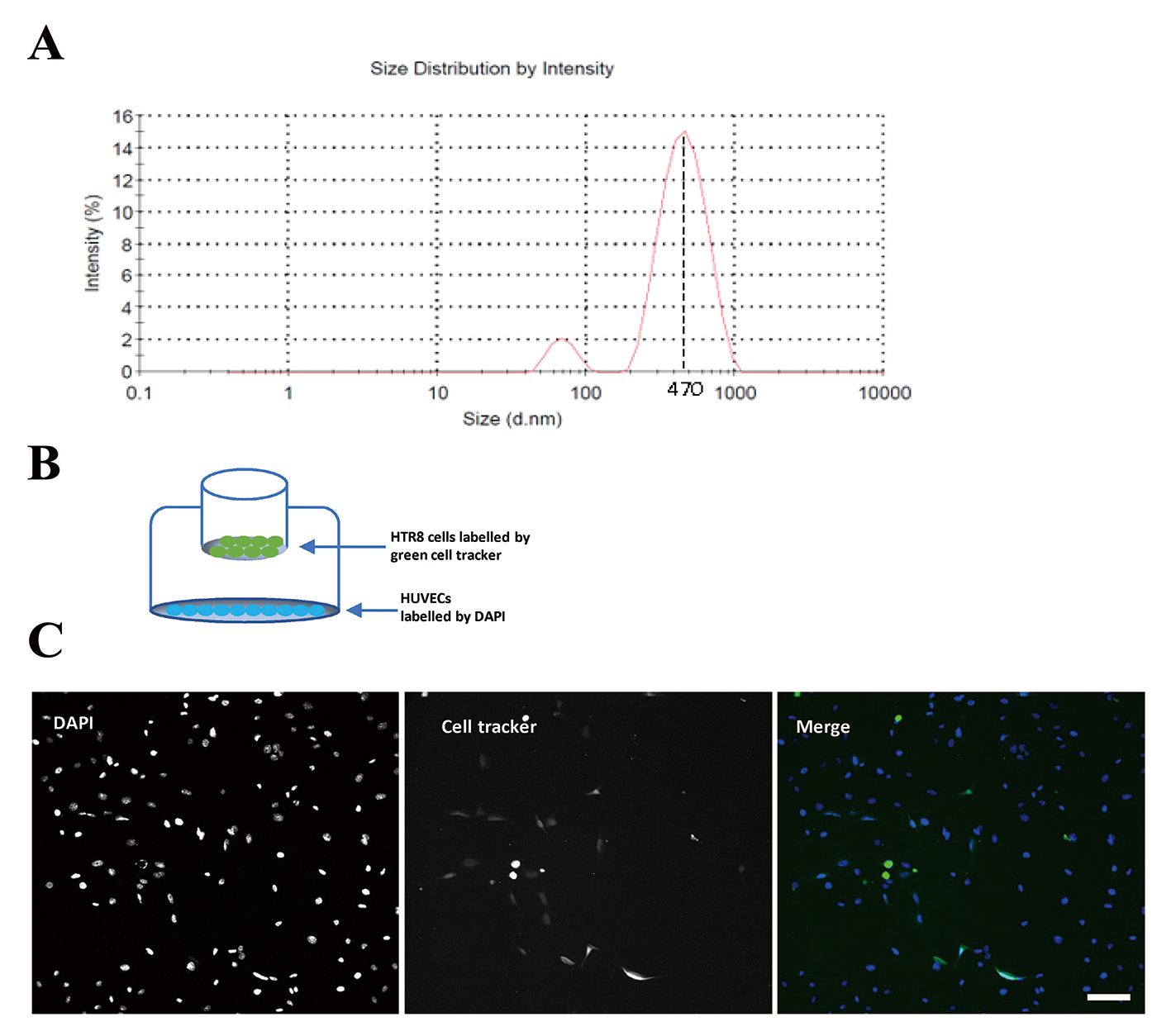

Supplement: Supplementary file 4 [file JCMM-24-4915-s004.tif]

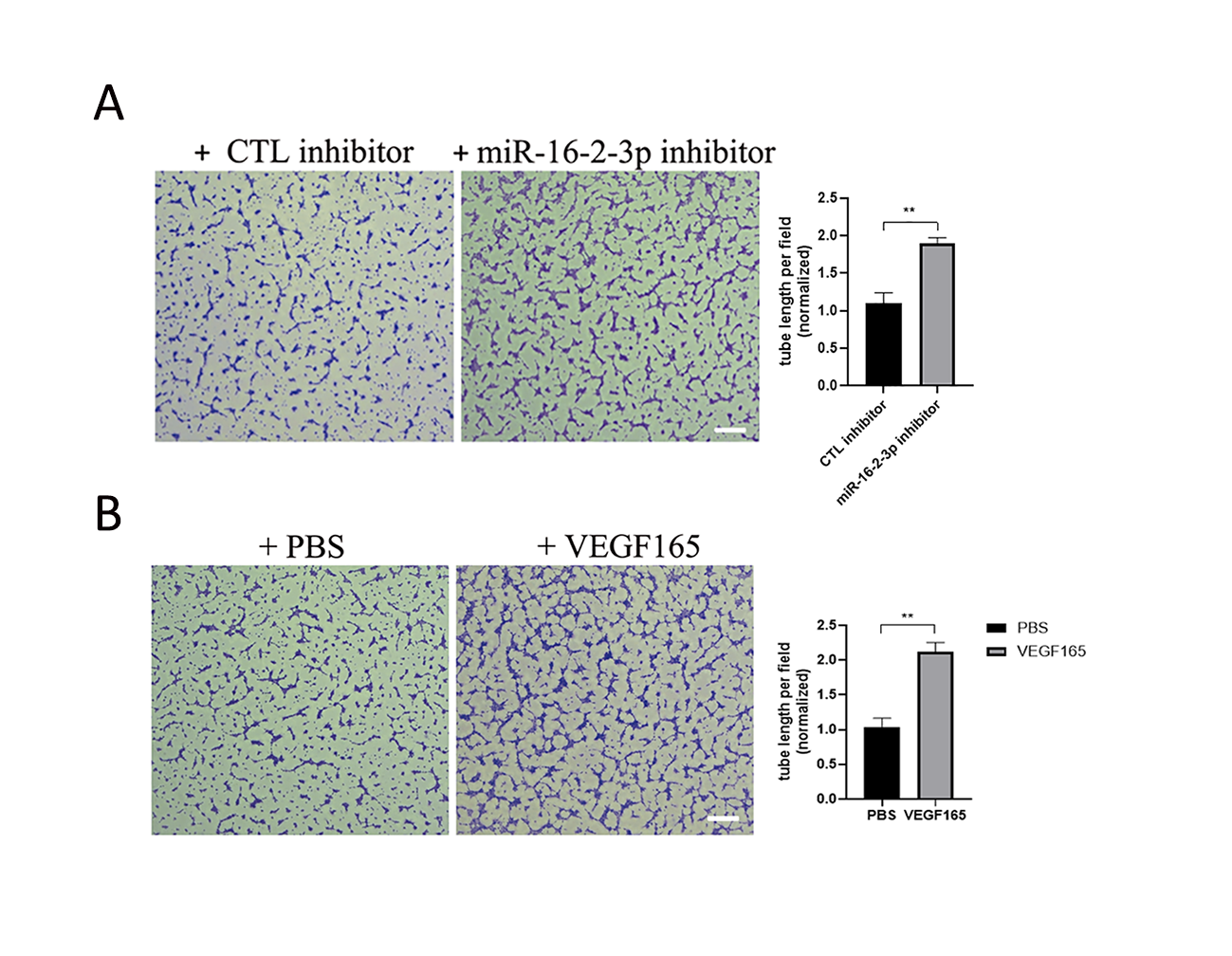

Supplement: Supplementary file 5 [file JCMM-24-4915-s005.tif]

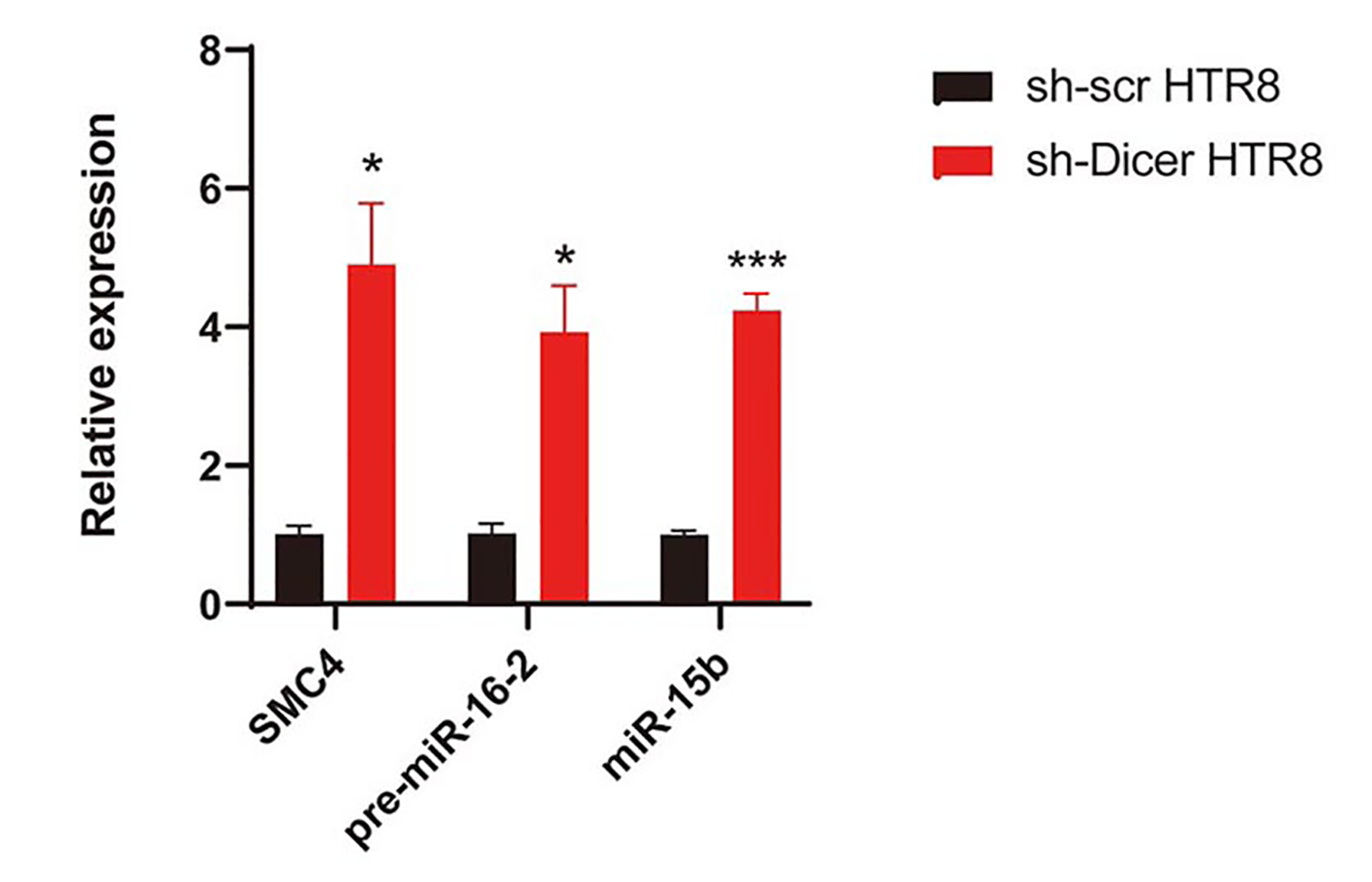

Supplement: Supplementary file 6 [file JCMM-24-4915-s006.tif]
