# Supplementary material for: Deficiency of DICER reduces the invasion ability of trophoblasts and impairs the pro‐angiogenic effect of trophoblast‐derived microvesicles
Source: J Cell Mol Med. 2020 Mar 21;24(9):4915–30. doi: 10.1111/jcmm.14917 (PMC7205818; doi:10.1111/jcmm.14917)
Supplement: Supplementary file 7 [file JCMM-24-4915-s007.docx]

Table.1 Clinical characteristics of normal and severe preeclamptic pregnancies.

| Characteristics | Control (n=8) | PE (n=6) | sPE (n=6) |  |
| --- | --- | --- | --- | --- |
| **Characteristics** | **Control (n=8)** | **PE (n=6)** | **sPE (n=6)** | **P Value** |
| maternal age (year) | 30 (18-33) | 27 (23-33) | 29 (22-34) | n.s |
| Gestational age at delivery (week) | 38 (37-39) | 32 (29-36) | 30 (27-33) | n.s |
| onset gestational age (week) | none | 31 (28-34) | 28 (26-32) | * |
| Systolic bloos pressure (mmHg) | 111 (102-123) | 149 (140-157) | 177 (162-190) | *** |
| Diastolic bloos pressure (mmHg) | 76 (60-85) | 95 (90-100) | 112 (105-120) | *** |
| Proteinuria (g/24h) | normal | 2.1 (1.5-2.6) | 4.3 (3.3-5.2) | *** |
| Neonatal birth weight (g) | 3401 (3130-3560) | 3193 (2950-3470) | 2288 (1440-3230) | *** |
