# Supplementary material for: Deficiency of DICER reduces the invasion ability of trophoblasts and impairs the pro‐angiogenic effect of trophoblast‐derived microvesicles
Source: J Cell Mol Med. 2020 Mar 21;24(9):4915–30. doi: 10.1111/jcmm.14917 (PMC7205818; doi:10.1111/jcmm.14917)
Supplement: Supplementary file 8 [file JCMM-24-4915-s008.docx]

Table 2. The primer sequences for RT-PCR and PCR

| **Gene** | **primer sequence** |
| --- | --- |
| GAPDH | F 5'-TGCACCACCAACTGCTTAGC-3' |
|  | R 5'-GGCATGGACTGTGGTCATGAG-3' |
| COL1A2 | F 5'-AACGGTGATAAAGGTCATGCTG-3' |
|  | R 5'-GTTTGCCAACTTCACCAGCG-3' |
| pre-miR-16-2 | F 5'-GTTCCACTCTAGCAGCACGT-3' |
|  | R 5'-GTCACACTAAAGCAGCACAGTA-3' |
| SMC4 | F 5'-CCCGTAAAGGCACCCAG-3' |
|  | R 5'-TTGGTCATTGCTGGAGGC-3' |
| DICER | F 5'-GTCGCTTGCTGGTGCCATTTAC-3' |
|  | R 5'-TCAGCCGGGCTAAATTTGGCAG-3' |
| VEGFA | F 5'-AGGGCAGAATCATCACGAAG-3' |
|  | R 5'-TACTCCTGGAAGATGTCCAC-3' |
| COL1A2 3'-UTR | F 5'-CCGTGTAATTCTAGATAACTGAAAGCTGAATCCTTCC-3' |
|  | R 5'-TCGGTCGACGGATCCTGAATCTGAAGAAGATTTGGGC-3' |
